# Influence of outdoor time on the spherical equivalent and axial length in childhood myopia: A meta‐analysis

**DOI:** 10.1111/aos.17478

**Published:** 2025-03-11

**Authors:** Clara Martinez‐Perez, Miguel Angel Sanchez‐Tena, José‐María Sánchez‐González, Cesar Villa‐Collar, Cristina Alvarez‐Peregrina

**Affiliations:** ^1^ ISEC LISBOA – Instituto Superior deEducação e Ciências, Lisbon, Portugal Lisbon Portugal; ^2^ Department of Optometry and Vision, Faculty of Optics and Optometry Universidad Complutense de Madrid Madrid Spain; ^3^ Department of Physics of Condensed Matter, Optics Area University of Seville Seville Spain; ^4^ School of Biomedical and Health Science Universidad Europea de Madrid Villaviciosa de Odón, Madrid Spain

**Keywords:** axial elongation, meta‐analysis, myopia prevention, outdoor activities, spherical equivalent

## Abstract

This meta‐analysis investigates the effectiveness of outdoor activities in reducing the onset of myopia in children and adolescents by analysing changes in axial elongation and spherical equivalent refractive error. Following PRISMA guidelines and registered in PROSPERO (CRD42024592971), the study included randomized controlled trials (RCTs) and observational studies. The eligibility criteria targeted children and adolescents aged 6 to 18 years with varying levels of outdoor exposure. Primary outcomes were axial elongation and spherical equivalent change. Studies were assessed for quality using GRADE and AMSTAR‐2 tools, and data were analysed using Review Manager 5.4, with random‐effects models applied when heterogeneity was significant. Fifteen studies (9 RCTs and 6 observational) were included, with a total of 16 597 participants. Outdoor activities significantly reduced or delayed the onset of myopia, with a mean axial length difference of −0.08 mm per year (95% CI: −0.09 to −0.07) and a spherical equivalent difference of 0.16 diopters per year (95% CI: 0.07 to 0.25). These differences were observed after 1 year of intervention and sustained for up to 3 years, with daily outdoor exposure ranging from 40 to 120 min. Heterogeneity was moderate to high, but sensitivity analyses confirmed the robustness of the results. While higher outdoor exposure appeared more effective in reducing myopia progression, the certainty of this evidence was rated as low due to suspected publication bias, as indicated by the GRADE analysis. Outdoor activities, especially with prolonged exposure to intense light, effectively reduce or delay the onset of myopia in children and adolescents. This study emphasizes the importance of light intensity in maximizing the benefits of outdoor interventions and highlights regional differences in effectiveness, suggesting that environmental factors play a significant role in the outcomes.

## 1 INTRODUCTION

Outdoor time and exposure to sunlight have gained interest in the prevention of myopia, an eye condition that has reached epidemic levels in Asia and Europe, affecting 80%–90% of teenagers in countries like South Korea and Singapore, and 50% in some European regions (Sankaridurg et al., 2021). Various studies suggest that outdoor activities reduce the risk of developing myopia, especially in children. This has led researchers to seek solutions based on increasing outdoor time and exposure to sunlight (Biswas et al., 2023; Chakraborty et al., 2022; He et al., 2022; Read et al., 2015). One of the most studied mechanisms is the intensity of outdoor light. It has been shown that high‐intensity light slows axial eye growth, which reduces the risk of myopia. Animal studies, such as those conducted in chickens, have demonstrated that intense light delays myopia‐induced axial changes (Biswas et al., 2023). Similarly, a study on children showed that those exposed to more sunlight experienced slower axial growth, indicating a significant correlation between sunlight and slower eye growth (Read et al., 2015).

In young and middle‐aged adults, exposure to intense light has also shown protective effects, such as choroidal thickening and reduced axial elongation (Chakraborty et al., 2022). In the study by He et al. (2022), it is suggested that daily exposure to sunlight for 120–150 min, with an intensity of at least 5000 lux per minute, can reduce the risk of myopia onset by 15%–24%. This effect is related to the release of dopamine, which regulates refractive development and axial eye growth (Ashby & Schaeffel, 2010; Feldkaemper & Schaeffel, 2013). Additionally, dopamine receptor antagonists, such as spironolactone, block this protective effect, highlighting the importance of dopamine in this process (Ashby & Schaeffel, 2010).

Light spectrum plays also a significant role in myopia control. Blue‐violet light (380–450 nm) has been shown to reduce axial elongation, primarily through mechanisms involving melanopsin signalling and retinal dopamine regulation (Thakur et al., 2021). In contrast, red light, specifically delivered through repeated low‐level red‐light therapy (RLRL) at 650 nm, has been associated with a thickening of the choroid and a slowing of axial elongation in controlled experimental settings (Jiang et al., 2022). It is important to emphasize that RLRL therapy involves specific exposure parameters that differ from those of everyday red light exposure, which limits the applicability of these findings to general environments. Both types of light may exert their effects through the release of nitric oxide, which enhances retinal blood flow and supports scleral remodelling, contributing to the observed reduction in myopia progression (Zhang & Zhu, 2022).

The circadian rhythm also influences myopia prevention, as light is key to its synchronization. Disruptions in these rhythms, caused by inadequate light exposure, may be related to the onset of myopia (Chakraborty et al., 2021; Mure, 2021).

An important aspect to consider is the difference between natural lighting and indoor artificial light. Natural light generally has higher intensity and a broader spectrum, which promotes accommodative relaxation and reduces ocular stress associated with prolonged near‐focus tasks (Bhandary et al., 2021; Lanca et al., 2019; Read et al., 2015; Wu et al., 2018). Furthermore, outdoor light stimulates the release of dopamine and melatonin, which regulate eye growth (Chakraborty et al., 2018; Chakraborty et al., 2021; Feldkaemper & Schaeffel, 2013; Kearney et al., 2017; Stone et al., 1989; Stone et al., 2013). A recent analysis of systematic reviews highlighted that early exposure to bright outdoor light reduces the relative risk of developing myopia by 24% to 46%, reinforcing the importance of this preventive approach in paediatric populations (Biswas et al., 2024; Dhakal et al., 2022; Karthikeyan et al., 2022; Lingham et al., 2020; Muralidharan et al., 2021; Wu et al., 2013).

Finally, the relationship between vitamin D synthesis, induced by ultraviolet radiation, and myopia has been debated. Some studies have found a negative correlation between serum levels of 25(OH)D3 and the incidence of myopia, although others suggest that this relationship may be more related to outdoor activity levels than to a direct protective effect of vitamin D (Chan et al., 2022; Pan et al., 2017; Tang et al., 2019). Although uncertainties remain, evidence suggests that maintaining adequate vitamin D levels could help prevent myopia by regulating scleral growth (McMillan, 2018; Mutti & Marks, 2011).

Several meta‐analysis studies address the impact of outdoor activities on myopia prevention, notably those by Deng and Pang (2019), Mei et al. (2024), Li et al. (2024) and Ho et al. (2019). The literature in this field is recent, with a growing focus in recent years due to the increasing prevalence of myopia, especially in children. These studies are generally of high quality, as many include RCTs and follow rigorous methodologies. They conclude that outdoor interventions significantly reduce the risk of myopia onset and slow its progression, as reflected by changes in spherical refraction and axial length.

However, these meta‐analyses also present biases, such as a lack of homogeneity in interventions and variation in follow‐up times, in addition to predominantly focusing on Asian populations. Most of the studies analyse information using random‐effects models and combine results using the mean difference and risk ratio. Commonly used variables are spherical equivalent refraction, axial elongation and myopia prevalence. Although most studies point in the same direction regarding the benefits of outdoor activities, some controversy remains. Certain studies have not found a significant relationship, which could be due to methodological, population, or intervention differences.

Our meta‐analysis aims to overcome these limitations by including additional analyses, such as GRADE to assess the quality of evidence, sensitivity analyses to test the robustness of the results, and publication bias assessments, which strengthen our conclusions regarding the effectiveness of outdoor interventions. Therefore, this study aims to evaluate the impact of outdoor time on the onset and progression of myopia by analysing axial length (AL) and spherical equivalent (SE) refraction in subgroups of myopes, non‐myopes and combined populations using randomized controlled trials. Additionally, this study assesses differences in outdoor exposure duration (hours/day) between myopes and non‐myopes using observational studies.

## 2 METHODS

### 2.1 Eligibility criterion

**FIGURE 1:**
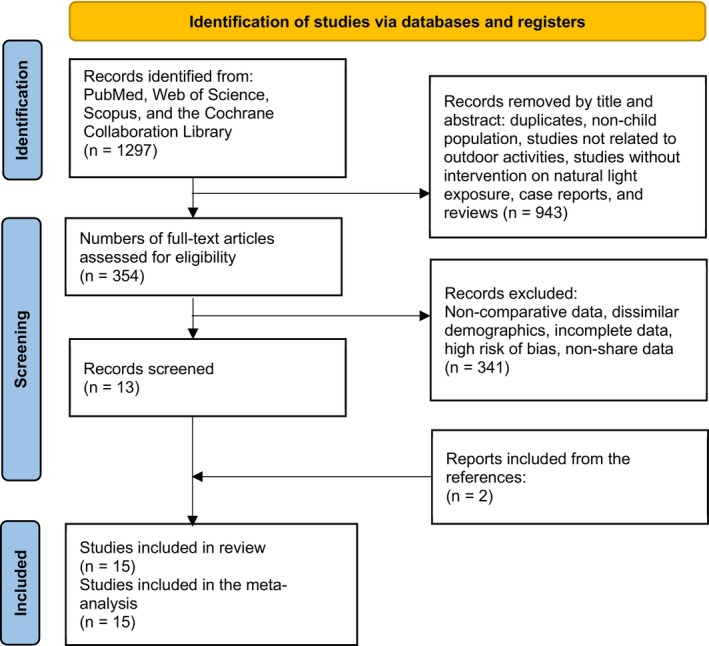
Study selection Flow chart (preferred reporting items for systematic reviews and meta‐analysis).

This meta‐analysis was registered in PROSPERO (CRD42024592971). The study followed PRISMA (Page et al., 2021) guidelines and AMSTAR‐2 (Shea et al., 2017) standards (Figure 1). The research question was formulated according to the PICOS strategy as follows: P (Population): Children and adolescents aged 6 to 18 years; I (Intervention): The intervention group consisted of those who participated in outdoor activities, with a minimum exposure to natural light varying across studies (e.g., 30 min to more than 14 h per week) measured either objectively or subjectively. For interventional studies, a minimum duration of 1 year of consistent outdoor exposure was required to evaluate its impact on AL and SE; C (Comparator): The comparator group included subjects who spent fewer hours outdoors and did not receive additional interventions related to outdoor exposure; O (Outcome): The primary outcome of interest was the onset and progression of myopia, evaluated using validated measures such as AL and SE. Myopia was defined as an SE of ≤−0.50 diopters, emmetropia was defined as an SE between −0.49 and +0.49 diopters, and hypermetropia was defined as an SE of ≥+0.50 diopters. These thresholds were consistently applied across the included studies to ensure uniformity and comparability in the analysis; S (Study Design): Observational studies, both prospective and retrospective and RCTs.

Exclusion criteria were applied to ensure the quality and comparability of included studies: Case reports: Studies presenting individual cases without a cohort design were excluded; Systematic reviews and literature reviews: These studies were excluded to avoid the inclusion of secondary analyses; Duplicates: Multiple publications reporting the same study were excluded to prevent data duplication; High risk of bias: Studies with significant risk of bias, such as those with inadequate methodology or poor design, were excluded; Unreported variables: Studies that did not report relevant variables, such as outdoor time or visual outcomes, were excluded; Non‐comparable data: Studies with data that could not be compared or pooled with those of other included studies were excluded; Incomplete data: Studies with insufficient or incomplete data to assess the relationship between outdoor activities and SE were excluded.

### 2.2 Information sources

An extensive literature review was conducted using several databases: PubMed, Web of Science and Scopus. A systematic and rigorous methodology was employed to search for relevant studies, without imposing specific time or language limits. Additionally, a thorough review of the bibliographic references of the studies selected in the first phase of the search was carried out to identify additional studies that may have been missed in the initial search.

### 2.3 Search methods for identification of studies

We used the following search terms to query all trial registries and databases: (‘outdoor’ OR ‘outside’ OR ‘outdoor activity’ OR ‘sunlight’) AND (‘myopia’ OR ‘nearsightedness’ OR ‘short sight’ OR ‘refractive error’) AND (‘child’ OR ‘children’ OR ‘paediatric’ OR ‘youth’ OR ‘adolescent’ OR ‘teenage’) AND (‘time’ OR ‘hour*’ OR ‘minute*’) (Appendix S1). Two reviewers independently agreed on the selection of eligible studies and reached a consensus on which to include.

### 2.4 Data extraction and data items

Two authors independently reviewed the data extracted from the studies. If consensus was not reached, a third author was consulted to complete the data extraction form. Basic characteristics of the articles were collected, including study name, period, follow‐up, region, study type, sample size, ethnicity and participant age. The main comparable variables were SE (defined as ≤−0.50 D for myopia, measured using cycloplegia or an open‐field autorefractometer) and AL (measured using light‐based optical biometry methods). In addition, it was assessed whether the studies were randomized and whether conflicts of interest and funding were reported (Appendix S2).

### 2.5 Risk of bias

#### 2.5.1 Randomized controlled trials (RCTs)

The methodological quality and risk of bias of the included RCTs were independently assessed by two reviewers using the Cochrane Collaboration's risk of bias tool (Review Manager 5.4 software). This tool systematically evaluates six key domains related to bias: random sequence generation, allocation concealment, blinding of participants and personnel, blinding of outcome assessment, incomplete outcome data and selective reporting. For each domain, explicit pre‐established criteria were used to assign ratings of low, high, or unclear risk of bias. Studies were excluded if they exhibited a high risk of bias across all six domains, as this was considered indicative of fundamental methodological flaws. However, studies with a high risk of bias in fewer than six domains were included if they provided essential data and their inclusion did not significantly affect the robustness of the overall analysis, as confirmed by sensitivity analyses.

#### 2.5.2 Non‐randomized studies

The methodological quality of the observational studies was independently assessed by two reviewers using the Methodological Index for Non‐Randomized Studies (MINORS) criteria (Slim et al., 2003). This instrument includes items to evaluate key methodological aspects such as study design, patient selection, outcome measures and follow‐up. For non‐comparative studies, only the first 8 items of the scale were evaluated, with a maximum possible score of 16 points. For comparative studies, all 12 items were assessed, with a maximum possible score of 24 points. Scores range from 0 to 16 for non‐comparative studies and from 0 to 24 for comparative studies. For non‐comparative studies, those with a score of 0–4 were considered to be of very low quality, 5–7 of low quality, 8–12 of moderate quality, and 13–16 of high quality. Comparative studies with scores of 0–6 were considered to be of very low quality, 7–10 of low quality, 11–15 of moderate quality, and 16–24 of high quality. Any discrepancies in the quality assessment between the two reviewers were discussed to reach a consensus.

### 2.6 Assessment of results

Mean differences (MD) and 95% confidence intervals (CI) were estimated for continuous variables measured on the same scale. Standardized mean differences (SMD) were used to account for different scales reported in disparate units. For dichotomous variables, odds ratios (OR) were calculated. Heterogeneity was assessed using the *I* ^2^ statistic, with values below 25%, between 25% and 50% and above 50% indicating low, moderate and high heterogeneity, respectively. A fixed‐effects model was used when no significant heterogeneity was observed (>50%). Incomplete data reported in the studies were addressed following the methodological guidelines of the Cochrane Handbook (Higgins et al., 2019). The statistical software Review Manager 5.4.1 was used for all analyses.

### 2.7 Publication bias

A funnel plot analysis was conducted using Review Manager 5.4.1 to assess potential publication bias. Asymmetry in the funnel plot may suggest publication bias due to the non‐publication of smaller studies with null or inconclusive results.

### 2.8 Additional analyses

Subgroup analyses were conducted to examine differences in outcomes based on several factors, including refractive status (myopes vs. non‐myopes), levels of exposure to natural light and duration of outdoor interventions.

Sensitivity analysis was performed by sequentially removing the studies with the highest weight in each subgroup to verify the robustness of the results and evaluate the impact of excluding individual studies on the overall conclusions. All comparisons were conducted using Review Manager 5.4.1, and a random‐effects model was used when heterogeneity was significant.

Additionally, the GRADE approach was employed to assess the certainty of the evidence, considering factors such as the risk of bias, inconsistency and publication bias (Guyatt et al., 2013).

## 3 RESULTS

### 3.1 Study selection

**FIGURE 2:**
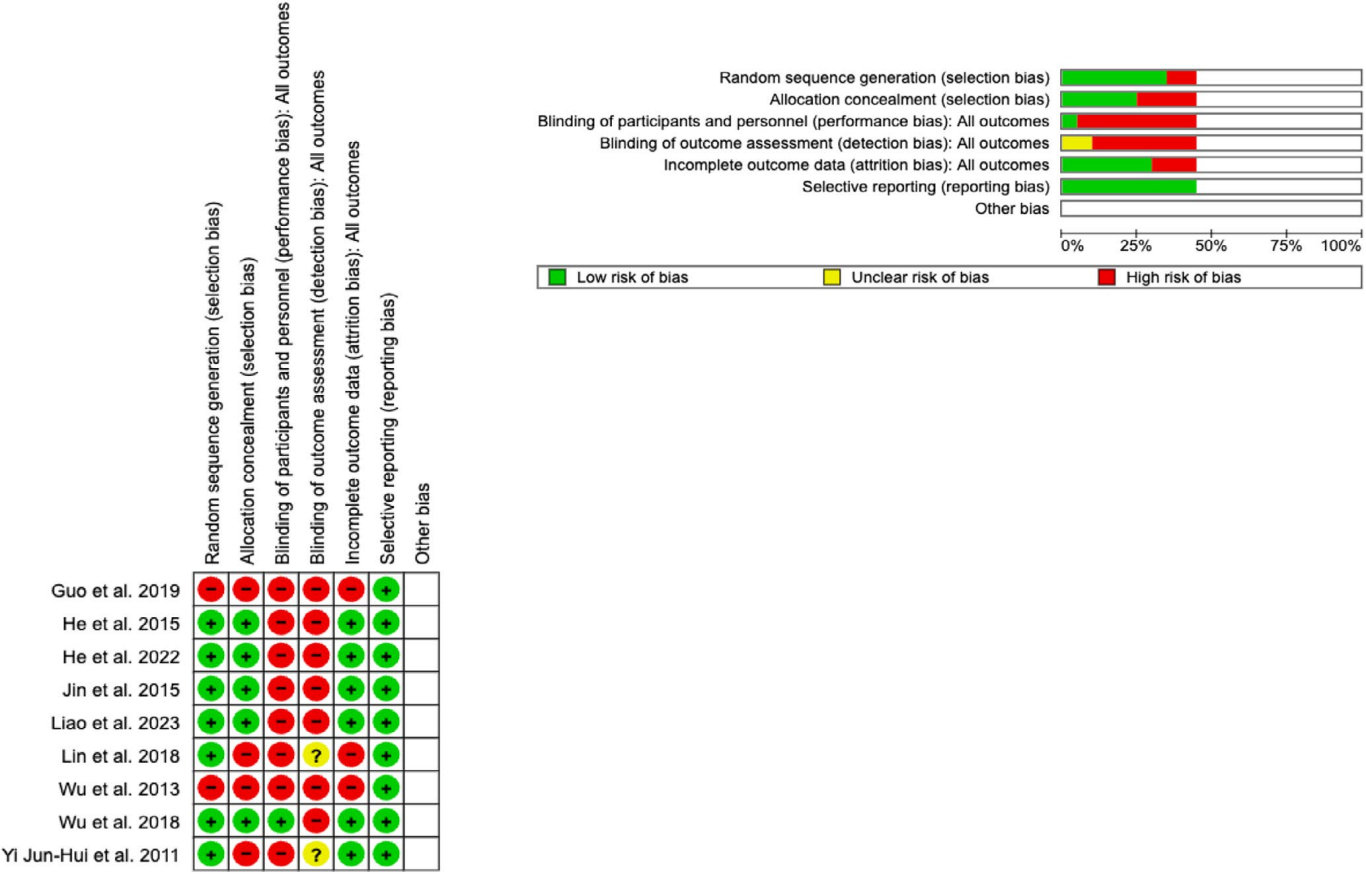
Risk of bias assessment (green = low risk; red = high risk; yellow = unknown) of nine RCTs.

The initial search yielded 1297 articles (Figure 1). After removing duplicates, studies not focused on children or adolescents, studies unrelated to outdoor activities or natural light exposure, case reports and reviews based on titles and abstracts, 943 studies were excluded. The remaining 354 articles underwent a detailed review. After reviewing the full text of these articles, 341 studies were excluded for not meeting inclusion criteria, not reporting comparable variables, having a high risk of bias, or containing incomplete data. The results of the risk of bias assessment are presented in Figure 2, and the justification for each criterion is provided in Appendix S3. Finally, 13 studies were included in the analysis. Two additional studies were identified from the references of the articles included. Therefore, 15 studies were included in the meta‐analysis (Atowa et al., 2020; Dhakal et al., 2024; French et al., 2013; Gopalakrishnan et al., 2023; Guo et al., 2019; He et al., 2015; He et al., 2022; Jin et al., 2015; Jones et al., 2007; Liao et al., 2023; Lin et al., 2018; Wen et al., 2020; Wu et al., 2013; Wu et al., 2018; Yi & Li, 2011).

### 3.2 Study characteristics

Table 1 summarizes the characteristics of nine interventional studies on outdoor activities for myopia prevention, including study region, age, study type, follow‐up duration, sample sizes and intervention details, involving 9125 participants with sample sizes ranging from 41 to 1993 subjects. It also indicates whether each study evaluated ‘myopic shift’ and ‘axial elongation’, reported as ‘Yes’ or ‘No’. Five studies were RCTs and four were prospective studies. The outdoor intervention consisted of activities such as outdoor exercise or daytime recess, with exposure times ranging from 30 min to 14 h per week. AL and SE were the main outcomes evaluated in all studies. Regarding randomization, five studies were randomized and reported significant differences in the onset and progression of myopia between the intervention and control groups, while prospective studies showed variability in their results.

**TABLE 1:** Baseline characteristics of the nine interventional studies on outdoor activities for Myopia prevention.

Study	Region	Race	Age	Type of study	Follow‐up (years)	n intervention/control group	Comparative groups	Outdoor exposure measurement	Randomized (yes/No)	Myopic shift (yes/No)	Axial length elongation (yes/No)
Guo et al. (2019)	Rural Beijing, China	Chinese	6–7	Prospective	1	157/216	Intervention group: 30 min of outdoor jogging per day; Control group: No additional outdoor time	Questionnaires (subjective)	No	Yes	Yes
He et al. (2015)	Guangzhou, China	Chinese	6–7	RCT	3	952/951	Intervention group: 40 min of outdoor activity per day during school time; Control group: usual activity patterns	Questionnaires (subjective)	Yes	Yes	Yes
He et al. (2022)	Shanghai	Chinese	6–9	RCT	2	Test I: 239; Test II: 1929/Control: 2037	Test I: +40 min/day; Test II: +80 min/day; Control: Habitual outdoor time (106 ± 27 min/day)	Wearable light sensor and questionnaires (objective + subjective)	Yes	Yes	Yes
Jin et al. (2015)	Northeast China	Chinese	6–14	Prospective	1	1993/1528	Intervention Group: 40 min per day; Control Group: No additional outdoor time	Questionnaires (subjective)	Yes	Yes	Yes
Liao et al. (2023)	Chengdu, China	Chinese	6–7	RCT	1	101/100	Outdoor exercise group: 60 min, 3 times a week; Outdoor control group: no additional outdoor exercise, only outdoor time	Protocol‐based intervention	Yes	Yes	No
Lin et al. (2018)	Wenzhou, China	Chinese	6–9	RCT	1	Test 1: 357; Test 2: 353/Control: 366	Test group 1: 7 h/week; Test group 2: 14 h/week; Control group: 3 h/week	Protocol‐based intervention	Yes	Yes	Yes
Wu et al. (2013)	Southern Taiwan	Chinese	7–11	Prospective	1	333/238	Intervention Group: 80 min/day outdoor during recess (6.7 h/week), Control Group: no additional outdoor time during recess	Questionnaires (subjective)	No	Yes	No
Wu et al. (2018)	Taiwan	Chinese	6–7	RCT	1	267/426	Intervention group: 11 h per week; Control group: usual recess time	Light meters (objective) + activity diaries (subjective)	Yes	Yes	No
Yi and Li (2011)	Hunan, China	Chinese	7–11	Prospective	2	41/39	Intervention Group: 14–15 h/week; Control Group: 6.2 ± 1.6 h/week	Questionnaires (subjective)	Yes	Yes	No

*Note*: Myopic shift: An increase in the degree of myopia, measured by a decrease in spherical equivalent.

Abbreviation: NR, Not reported.

Table 2 summarizes the characteristics of the six observational studies on the association between outdoor activities and the onset and progression of myopia. Six observational studies conducted in regions such as Africa, India, Australia and the United States, with a total of 7472 participants, were included. None of these studies included randomisation. Observational studies found that non‐myopic children tended to spend more time outdoors than myopic children, but results regarding AL and SE were more heterogeneous. The quality of the studies was evaluated as moderate in most cases (Table 3).

**TABLE 2:** Baseline Characteristics of the Six Observational Studies on the Association Between Outdoor Activities and myopia onset.

Study	Region	Race	Age	Type of study	Follow‐up (years)	n	Comparative groups	Outdoor exposure measurement	Randomized (yes/No)	Myopic shift (yes/No)	Axial length elongation (yes/No)
Atowa et al. (2020)	Aba, Nigeria	African	8–15	Cross‐sectional	NR	1197	Myopic children: 4.1 ± 1.9 h/week, Non‐myopic children: 8.4 ± 2.6 h/week	Questionnaires (subjective)	No	No	No
Dhakal et al. (2024)	India	Indian	9–15	Longitudinal observational study	1	143	Myopes: 44 min/day, Non‐myopes: 51 min/day	Validated wearable light tracker (objective)	No	No	No
French et al. (2013)	Sydney, Australia	Mixed (primarily European Caucasian and East Asian)	6–12	Longitudinal cohort study	Younger cohort: 6–7 years; Older cohort: 12 years	2103	Younger cohort: 16.3 h/week (myopic) vs. 21.0 h/week (non‐myopic); Older cohort: 17.2 h/week (myopic) vs. 19.6 h/week (non‐myopic)	Questionnaires (subjective)	No	No	No
Gopalakrishnan et al. (2023)	Tamil Nadu	South India	12–16	Cross‐sectional	NR	3429	Myopes: 2.27 h/day, Non‐myopes: 2.38 h/day	Questionnaires (subjective)	No	No	No
Jones et al. (2007)	United States	Mixed (primarily Caucasian)	8–9	Prospective observational study	5	514	Non‐myopic children: 11.65 h/week of outdoor activity; Future myopic children: 7.98 h/week of outdoor activity	Questionnaires (subjective)	No	No	No
Wen et al. (2020)	Changsha, Hunan, China	Chinese	10.13 ± 0.48	Cross‐sectional study	1 week	86	Myopic children: 0.68 h/day; Non‐myopic children: 1.02 h/day	Clouclip device (objective)	No	No	No

*Note*: Myopic shift: An increase in the degree of myopia, measured by a decrease in spherical equivalent.

Abbreviation: NR, Not reported.

**TABLE 3:** Assessment of the quality of studies through the Methodological Index for Non‐Randomized Studies (MINORS).

Study	Clearly stated aim	Consecutive patients	Prospective collection data	Endpoints	Assessment endpoint	Follow‐up period	Loss less than 5%	Study size	Adequate control group	Contemporary group	Baseline control	Statistical analyses	MINORS	Type	Quality level
Atowa et al. (2020)	2	0	1	2	2	0	3	2	–	–	–	–	12	Non‐comparative	Moderate Quality
Dhakal et al. (2022)	2	2	1	2	2	2	2	2	–	–	–	–	15	Non‐comparative	High Quality
French et al. (2013)	2	2	2	2	2	2	2	2	2	2	2	2	24	Comparative	High Quality
Gopalakrishnan et al. (2023)	2	0	0	2	2	0	0	2	–	–	–	–	8	Non‐comparative	Low Quality
Jones et al. (2007)	2	2	1	2	2	2	2	2	–	–	–	–	15	Non‐comparative	High Quality
Wen et al. (2020)	2	0	1	2	2	0	0	2	–	–	–	–	9	Non‐comparative	Moderate Quality

### 3.3 Outcomes

**FIGURE 3:**
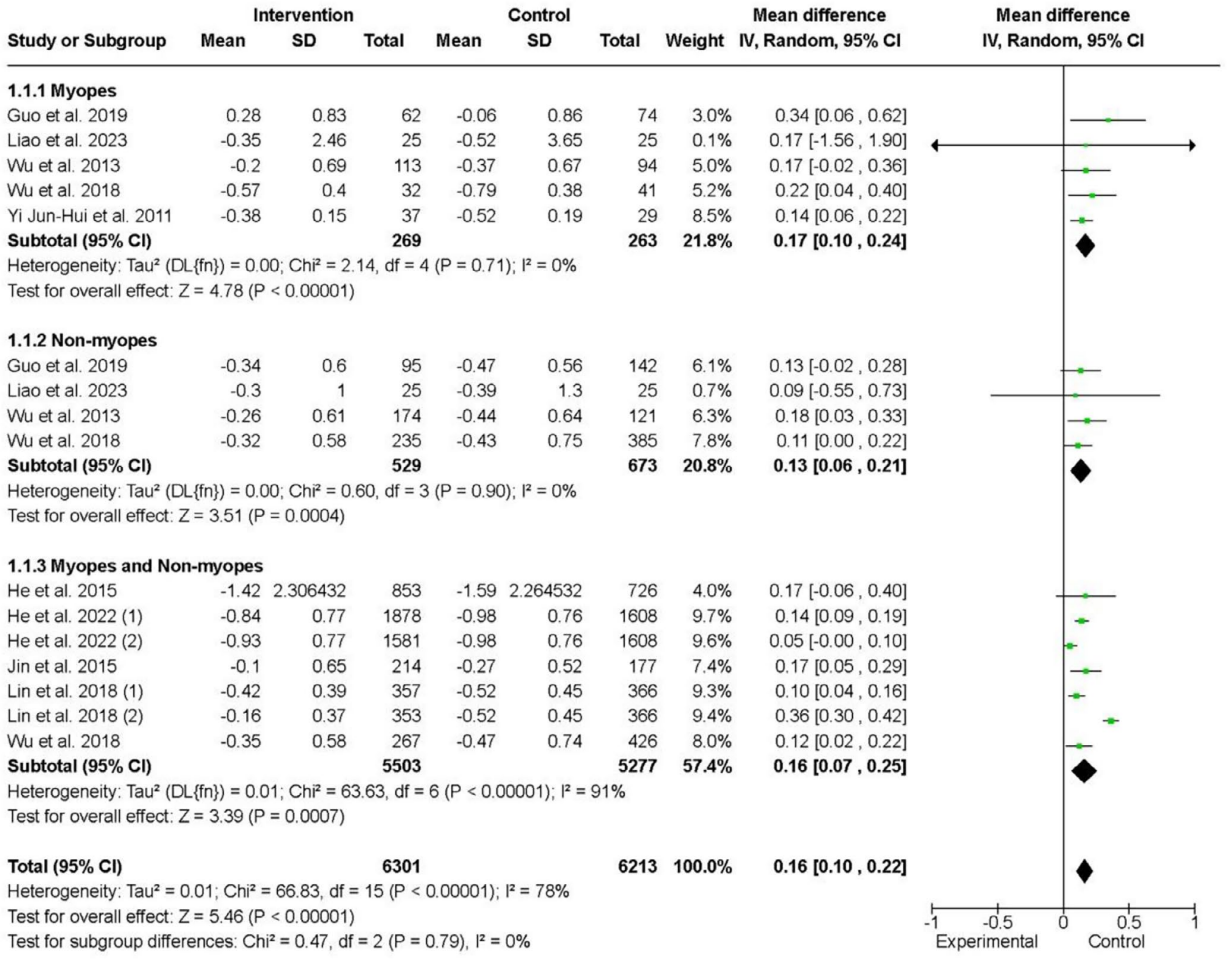
Effect of outdoor interventions on the spherical equivalent change (D) in myopic, non‐myopic children, and a combination of both. Numbers (1) and (2) indicate different tests within the RCTs. In the He et al., Number 1 added 40 min of outdoor time, while number 2 added 80 min. In Lin et al., Number 1 spent 7 h per week outdoors, while Number 2 spent 14 h per week outdoors.

Figure 3 presents the pooled results from RCTs on outdoor activities for myopia prevention, focusing on changes in SE. The analysis considered three categories: myopes, non‐myopes and a combined group. In the myopic subgroup, five studies with a total of 263 participants reported standardized mean differences (SMD) favouring outdoor interventions, with a mean difference of 0.17D (95% CI: 0.10 to 0.24) and low heterogeneity (*I* ^2^ = 0%). For the non‐myopic subgroup, four studies including 529 participants showed a mean difference of 0.13D (95% CI: 0.06 to 0.21), also with low heterogeneity (*I* ^2^ = 0%). In the combined subgroup, seven studies with 5503 participants reported a mean difference of 0.16D (95% CI: 0.07 to 0.25), but heterogeneity was moderate (*I* ^2^ = 91%). Overall, the pooled population of 4239 participants demonstrated a mean difference of 0.16D (95% CI: 0.10 to 0.22), with consistent findings across most studies and low heterogeneity in the majority of subgroups.

**FIGURE 4:**
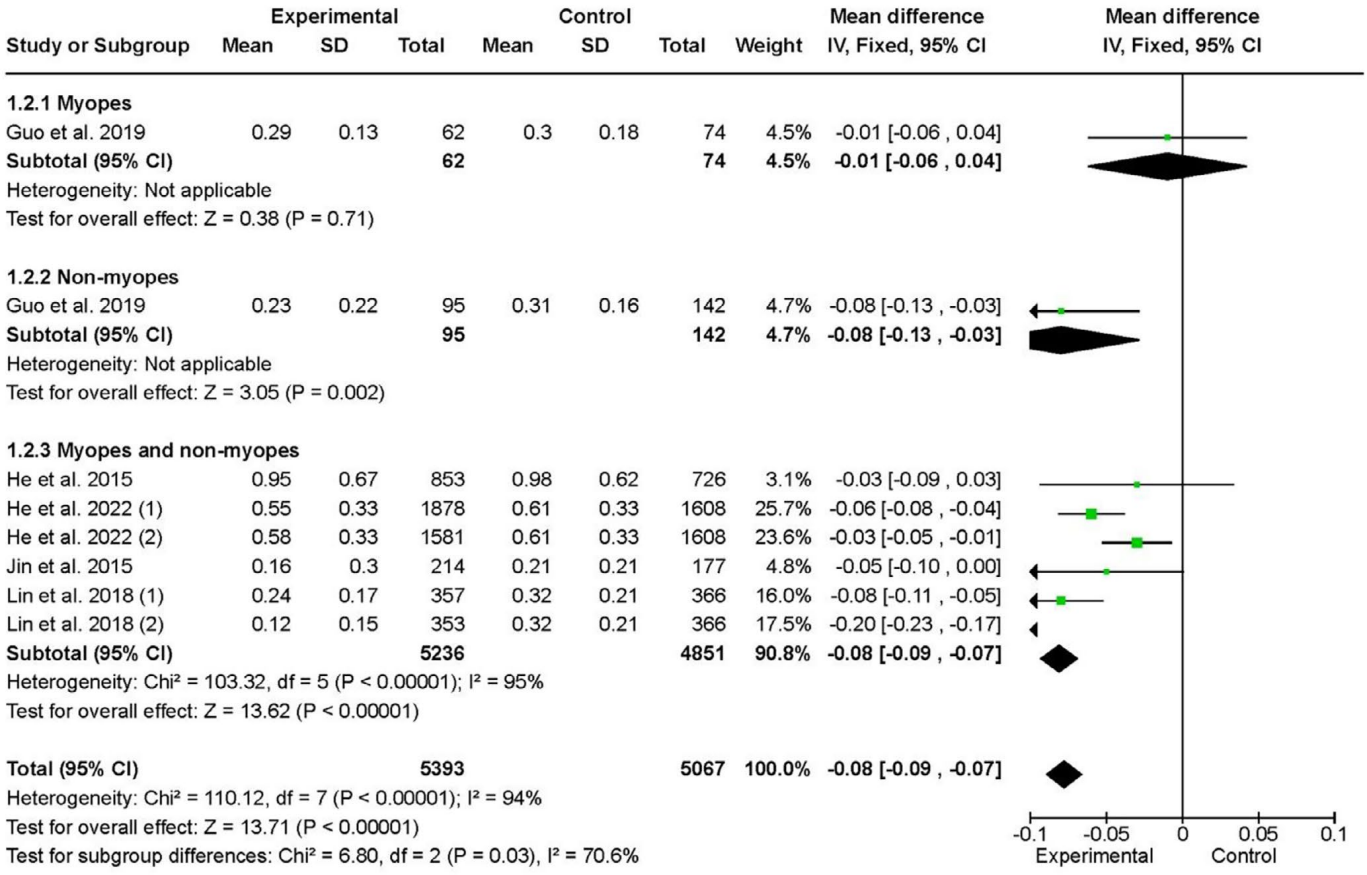
Effect of outdoor interventions on axial length evolution between initial and final measurements in myopic, non‐myopic children and a combination of both. Numbers (1) and (2) indicate different tests within the RCTs. In the He et al., Number 1 added 40 min of outdoor time, while number 2 added 80 min. In the Lin et al., Number 1 spent 7 h per week outdoors, while Number 2 spent 14 h per week outdoors.

Figure 4 analyzes changes in axial length (AL) across the same subgroups, based exclusively on RCTs. In the myopic subgroup, one study with 138 participants reported no significant difference between intervention and control groups, with a mean difference of −0.01 mm (95% CI: −0.06 to 0.04, *p* = 0.71), and no heterogeneity was reported. In the non‐myopic subgroup, two studies with 237 participants showed a significant reduction in AL among children in the intervention group, with a mean difference of −0.08 mm (95% CI: −0.13 to −0.03, *p* = 0.002), and no heterogeneity. In the combined subgroup, six studies including 8329 participants found a significant reduction in AL in the intervention group, with a mean difference of −0.08 mm (95% CI: −0.09 to −0.07, *p* < 0.0001), although heterogeneity was high (*I* ^2^ = 95%).

**FIGURE 5:**
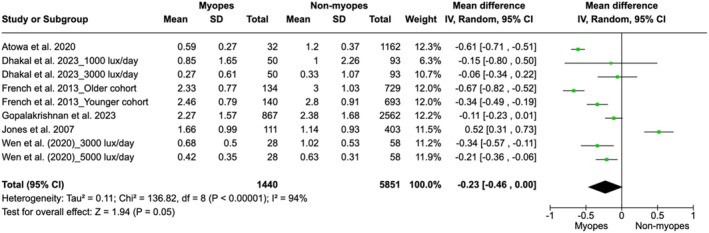
Mean difference in the number of hours/day of outdoor exposure between myopic and non‐myopic children.

Based on observational studies, Figure 5 shows the difference in daily outdoor exposure (measured in hours per day) between myopic and non‐myopic children. The presented studies evaluated the average duration of natural light exposure and its impact on the onset and progression of myopia. A negative mean difference indicates that non‐myopic children had greater outdoor exposure compared to myopic children. Overall, the mean difference between myopic and non‐myopic groups was −0.23 h/day (95% CI: −0.46 to 0.00), indicating a trend toward greater exposure in non‐myopic children. However, the overall value did not reach statistical significance (*p* = 0.05). Heterogeneity among the studies was high (*I* ^2^ = 94%), indicating considerable variability in the results of the included studies.

### 3.4 Sensitivity analysis

**FIGURE 6:**
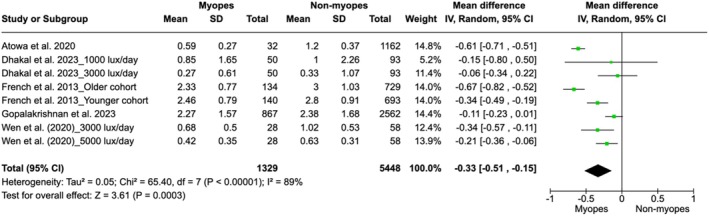
Sensitivity analysis about the association between time spent outdoors and myopia progression in myopes and non‐myopes.

Since the study by Jones et al. (2007) demonstrated an effect direction contrary to that of other observational studies, it was excluded to perform a sensitivity analysis. Consequently, a significant alteration in the overall results was observed (Figure 6). The weighted mean difference between the myopic and non‐myopic groups was −0.33 h/day (95% CI: −0.51 to −0.15), indicating that non‐myopic children were exposed to more hours of daily sunlight compared to myopic children. The heterogeneity among the studies slightly decreased, from *I* ^2^ = 94% in the original analysis to *I* ^2^ = 89% after the removal of the study, suggesting a reduction in variability among the included studies.

The *Z*‐value for the overall effect was 3.61 (*p* = 0.0003), confirming the statistical significance of sunlight exposure in reducing the onset of myopia. This sensitivity analysis highlights the impact of individual studies on the pooled results and provides a more consistent estimate after the removal of this specific study.

### 3.5 Publication bias

**FIGURE 7:**
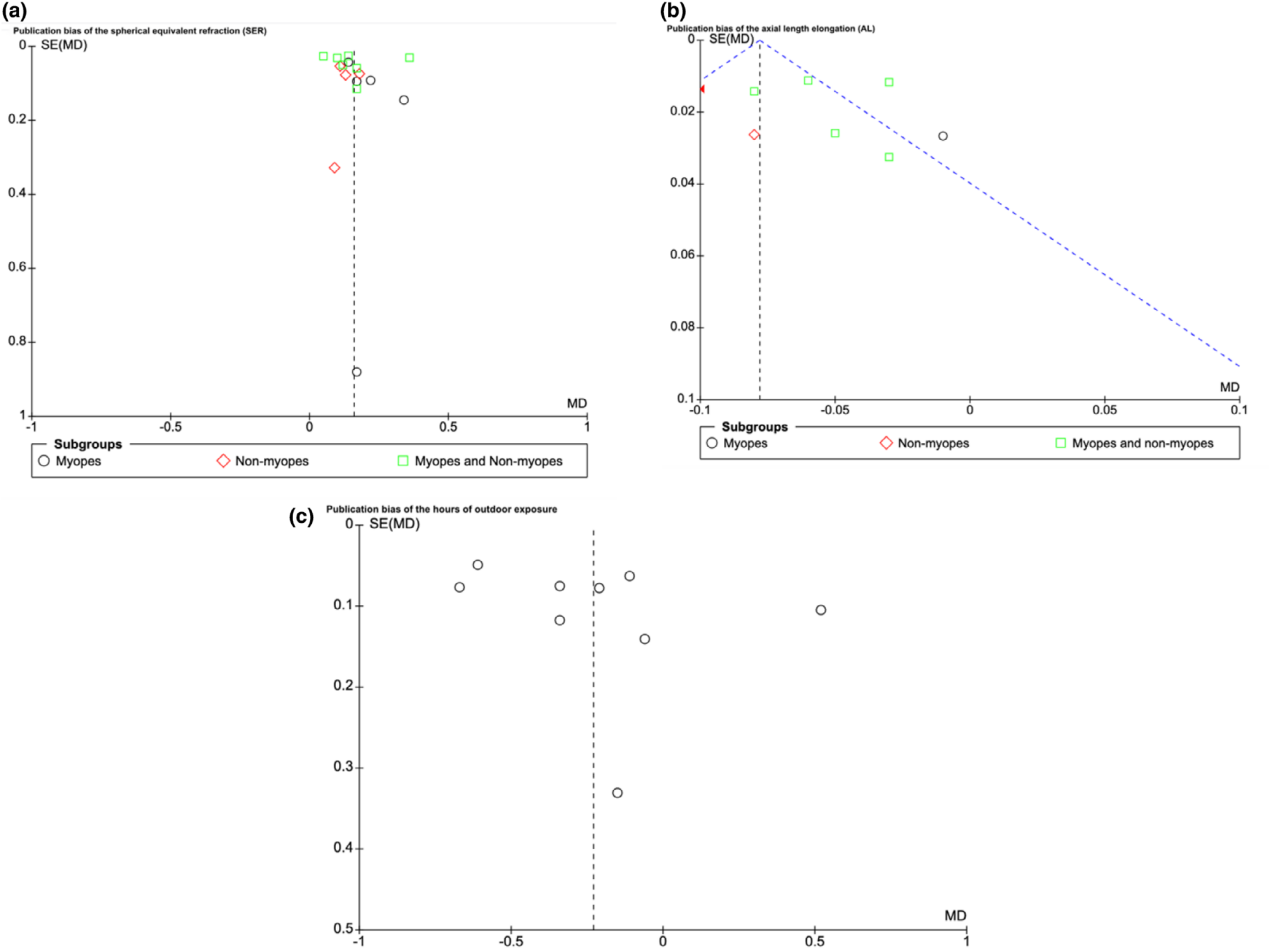
Funnel plot demonstrating no publication bias. The values were found to be in an acceptable range: (a) publication bias of the hours of outdoor exposure; (b) publication bias of the axial length elongation (AL); (c) publication bias of the spherical equivalent refraction (SER).

Publication bias was assessed using funnel plots, revealing no heterogeneity and publication bias across all complication outcomes, including axial elongation progression, refractive changes, and hours of outdoor exposure (Figure 7).

### 3.6 Grade

The GRADE (Grading of Recommendations, Assessment, Development and Evaluation) summary of the results for these three comparisons is shown in Table 4. There was moderate certainty for the change in SER, high certainty for axial length (AL), and low certainty for hours of outdoor exposure due to suspected publication bias.

**TABLE 4:** GRADE assessment of the quality of the evidence and the strength of the recommendations.

Certainty assessment							№ of patients		Effect		Certainty	Importance
№ of studies	Study design	Risk of bias	Inconsistency	Indirectness	Imprecision	Other considerations	[Intervención]	[Comparación]	Relative (95% CI)	Absolute (95% CI)		
*SER*												
9	Randomized trials	Serious^a^	Not serious	Not serious	Not serious	Publication bias strongly suspected strong association^b^	6301/10540 (59.8%)	6213/10540 (58.9%)	OR 0.16 (0.10 to 0.22)	403 fewer per 1000 (from 464 fewer to 349 fewer)	⨁⨁⨁◯ Moderate^a^, ^b^	IMPORTANT
*AL*												
5	Randomized trials	Serious^a^	Not serious	Not serious	Not serious	Strong association	5393/8486 (63.6%)	5067/8486 (59.7%)	OR −0.08 (−0.09 to −0.07)	732 fewer per 1000 (from 751 fewer to 713 fewer)	⨁⨁⨁⨁ High^a^	CRITICAL
*Hours*												
6	Non‐randomized studies	Not serious	Not serious	Not serious	Not serious	Publication bias strongly suspected strong association^b^	1440/5700 (25.3%)	5851/5700 (102.6%)	OR −0.23 (−0.46 to 0.00)	127 fewer per 1000 (from 80 fewer to –)	⨁⨁◯◯ Low^b^	CRITICAL

Abbreviations: CI, confidence interval; OR, odds ratio.

^a^ Risk of bias assessed with the Cochrane Collaboration's risk of bias tool (Review Manager 5.4 software).

^b^ Publication bias detected through funnel plot.

## 4 DISCUSSION

This meta‐analysis provides robust evidence that increased outdoor time significantly reduces the onset and progression of myopia in children and adolescents. The findings are particularly notable in terms of spherical equivalent and axial length, especially within non‐myopic and combined groups.

The included studies showed that greater exposure to natural light (more than 7 h per week) is associated with lower AL (−0.08 mm, 95% CI: −0.09 to −0.07) and SE (−0.16 D, 95% CI: 0.07 to 0.25). Longer interventions, particularly those exceeding 1 year, showed better outcomes, although not all studies found significant differences, which could be due to variability in study designs and geographical conditions. Studies conducted in Asia showed greater effectiveness compared to other regions, highlighting the influence of environmental and demographic factors. Observational studies such as Wu et al. (2018) and Dhakal et al. (2024) suggest that light intensity exceeding 10,000 lux is a critical determinant of the effectiveness of outdoor exposure in myopia prevention. However, in this meta‐analysis, light intensity was not evaluated as an independent variable due to the limited and inconsistent data available in the included RCTs. Therefore, while our findings support the recommendation for increased outdoor time, they also highlight the need for future research to investigate the independent contributions of light intensity, light spectrum and outdoor duration to myopia prevention. The risk of bias was moderate, being lower in RCTs and higher in observational studies. Some publication bias was detected, but sensitivity analyses confirmed the robustness of the results. However, several limitations should be considered when interpreting these findings. One of the main limitations of our study was the heterogeneity among the included articles, which limited the ability to conduct more detailed subgroup analyses and regressions. The studies included in this meta‐analysis showed considerable variation in the type and duration of outdoor exposure. While some studies focused on structured outdoor activities, such as supervised recess or school‐based programs, others examined general time spent outdoors during leisure hours. Structured activities may provide more consistent and controlled exposure to natural light compared to unstructured activities, which may vary significantly depending on individual behaviour and environmental factors. Furthermore, the duration of outdoor exposure was reported inconsistently, ranging from daily hours to weekly aggregates, making direct comparisons challenging. These differences underscore the need for standardized methodologies in future research to differentiate the type and duration of outdoor exposure and better evaluate their relative contributions to myopia prevention. Additionally, we did not stratify results by myopia severity, which could provide insights into the differential effects of outdoor exposure. The inconsistency in the literature, where many studies present varied results, further complicated direct comparisons. It is worth noting that in the study by Dhakal et al. (2024), the authors presented data using median and range rather than mean and standard deviation, complicating comparisons with other studies that used different statistical methods. This difference in data presentation limits the ability to integrate results uniformly into analyses, introducing possible variations in the final interpretation of results. The inclusion of non‐randomized studies introduces potential bias, which was mitigated through systematic assessment with the MINORS scale and sensitivity analyses. However, according to the GRADE framework, the certainty of evidence for hours of outdoor exposure is low, mainly due to publication bias and variability in study designs. This implies that the recommendation of 2 h of daily outdoor exposure should be interpreted with caution, as the evidence base for this specific threshold remains uncertain. While sensitivity analyses confirmed that excluding lower‐quality studies did not alter the results, the limitations in evidence strength must be acknowledged in the interpretation of findings. Future studies with standardized methodologies and better control of publication bias are needed to strengthen the conclusions. Finally, the use of standardized mean differences (SMD) addressed disparities in measurement methods across studies, ensuring a consistent approach to data synthesis despite differences in how outdoor exposure was measured. Regarding the spherical equivalent, our findings indicate an average difference of 0.16 diopters in children, which is consistent with what was reported by Ho et al. (2019), where they found a mean reduction of 0.15 diopters per year in spherical equivalent refractive error in children who increased their outdoor exposure time. Our study emphasized that younger children (aged 6–9) benefit more from these interventions, suggesting that early interventions may be more effective in delaying the onset of myopia. Similarly, Xiong et al. (2023), in a study conducted in urban China, observed a decrease of 0.19 diopters in children who participated in outdoor activities for more than 14 h per week, supporting the idea that there is a dose–response relationship between outdoor time and myopia progression. However, not all studies show such a significant reduction in SE; Chakraborty et al. (2022) conducted an analysis in European populations and found no statistically significant differences in SE in those regions with lower sunlight exposure during winter. The authors suggest that the results are influenced by sunlight intensity at different latitudes, highlighting the need to tailor recommendations according to geographic and climatic conditions. This difference in results underscores the importance of environmental and climatic conditions, which may influence the effectiveness of outdoor time interventions. It may also reflect differences in sunlight intensity, as several studies suggest that high‐intensity light (>10 000 lux) is crucial for obtaining maximum benefit in terms of myopia prevention (Dhakal et al., 2024). This may suggest that non‐myopic children had significantly greater sunlight exposure compared to myopic children or combined groups.

Regarding AL, our findings, which show an average difference of −0.08 mm, are comparable to those reported by Deng and Pang (2019), who found in a meta‐analysis of five clinical trials that outdoor activities result in a lower AL by an average of −0.03 mm of difference per year in the intervention group compared to the control group. Although this difference is more modest than reported in our study, it reinforces the idea that increasing outdoor time has a beneficial effect on eye growth, especially in terms of AL. The difference in results may be attributed to variability in the light intensity to which participants were exposed or the age of the children included in the studies, as exposure to intense light has been shown to be key in reducing AL. Ashby and Schaeffel (2010) also observed a significant decrease in AL (−0.08 mm) in Australian children who participated in a 12‐h‐per‐week outdoor school program. A possible explanation for the similarity in results between studies conducted in Asia and Australia could be the intensity of sunlight in these regions, which tends to be higher compared to Europe or the Middle East. However, it is important to exercise caution when generalizing these findings, as both Asia and Australia encompass vast and diverse geographical areas with significant variations in climate, altitude and seasonal light exposure. For instance, sunlight intensity in northern Australia differs substantially from southern regions, just as urbanized parts of Asia may experience different environmental conditions compared to rural areas. These differences highlight the need for more localized studies to confirm whether the relationship between sunlight exposure and myopia onset is consistent across different subregions (Bhandary et al., 2021, ‘Global Solar Atlas’, 2024). In fact, light intensity plays a key role in reducing SE, as highlighted by Wu et al. (2018) who reported greater efficacy in slowing axial growth in children exposed to intense sunlight (>10 000 lux). This finding suggests that not only time but also light quantity, is crucial for maximizing the benefit of outdoor interventions.

An additional mechanism that could further explain the progression of myopia in children who stay predominantly indoors is the increased accommodative demand imposed by near‐work activities under artificial lighting conditions. Indoor environments are not only characterized by reduced light intensity but also by limited visual distances, which require sustained accommodation. This prolonged accommodative effort can contribute to axial elongation, as the eye adapts to the visual demands of close work (Harb et al., 2023) highlighted that children who spend extended periods indoors may experience both a reduction in exposure to high‐intensity light and an increase in accommodative stress, both of which are associated with myopia progression. Therefore, the protective effect of outdoor activities may extend beyond exposure to natural light, as it also allows the eyes to relax by focusing on distant objects, reducing accommodative strain. This insight underscores the multifactorial nature of myopia development and highlights the importance of considering accommodative mechanisms when designing interventions to mitigate myopia progression.

In terms of hours of outdoor exposure, several studies confirm the effectiveness of spending more time outdoors. Wu et al. (2018) found that 11 h per week of outdoor activities were associated with a 30% reduction in the onset of myopia in Taiwanese children. The authors highlighted that the intervention was more effective in those children who spent at least 2 h outdoors during school recess, suggesting that integrating these activities into the school routine can maximize the benefit. Rose et al. (2008) also observed that children who spent more than 2 h per day outdoors had a significantly lower rate of myopia.

However, differences in the methods used to measure outdoor exposure, ranging from subjective questionnaires to objective light sensors, may influence the comparability of these results. Subjective methods, such as parent‐reported questionnaires, are prone to recall bias and overestimation, which may partially explain the variability in the reported benefits of outdoor exposure (Harb et al., 2023). In contrast, objective tools, such as light sensors, provide precise, real‐time data on both duration and intensity of light exposure, enabling more accurate assessments of its effects (Dhakal et al., 2022; Read et al., 2015). For instance, Dhakal et al. (2024) pointed out that light intensity is a key factor. In their study, children exposed to more than 10,000 lux of sunlight for at least 2 h a day had significantly lower progression and onset compared to those who only spent time outdoors under moderate light conditions. This finding suggests that, in addition to the number of hours, the intensity of natural light exposure is crucial in reducing SE.

Comparing our results with other meta‐analyses (Deng & Pang, 2019; Ho et al., 2019; Li et al., 2024; Mei et al., 2024), our findings align in that outdoor interventions result in significantly lower values of SE in children and adolescents. However, our meta‐analysis offers greater methodological robustness by using tools such as GRADE and AMSTAR‐2 to evaluate the quality of the evidence. These tools allowed us to ensure more solid conclusions by more comprehensively addressing the risk of bias, study consistency and the possibility of publication bias, which were not explored in detail in other studies. Additionally, while Ho et al. (2019) and Li et al. (2024) identified a dose–response relationship between outdoor time and lower values of SE, our analysis added a more precise focus, considering not only time spent outdoors but also sunlight intensity (>10 000 lux) as a key factor in maximizing benefits. This observation, which was not deeply analysed in previous studies, reinforces the idea that not only outdoor time but the quantity of light exposure is crucial in slowing SE. Another strength of our analysis is the inclusion of a greater number of studies and the performance of subgroup analyses, which allowed us to capture regional and geographical variations in more detail. For instance, we identified that interventions were more effective in Asia, likely due to higher sunlight intensity in this region, while studies in regions with lower sunlight exposure, such as Europe, showed reduced effectiveness. This ability to break down results by region highlights one of our main contributions, as previous analyses did not delve as deeply into these differences. For instance, studies conducted in Asia, such as He et al. (2015) and Jin et al. (2015), demonstrated greater effectiveness in reducing myopia progression compared to studies in Europe, as noted by Chakraborty et al. (2022). This regional difference can be attributed to variations in sunlight intensity, with studies like Wu et al. (2018) and Dhakal et al. (2024) emphasising that light intensities exceeding 10 000 lux are crucial for achieving optimal protective effects. Conversely, Chakraborty et al. (2022) noted reduced effectiveness in European regions, where sunlight exposure is lower, particularly during winter months. This ability to break down results by region highlights one of our main contributions, as previous analyses did not delve as deeply into these differences. Finally, compared to Deng and Pang (2019) and Mei et al. (2024), our results on the lower SE (0.16 diopters vs. −0.13 diopters/year in Deng and Pang (2019)) and AL (−0.08 mm vs. −0.09 mm in Mei et al., 2024) are consistent, but our sensitivity analyses and rigorous heterogeneity tests allowed us to identify the robustness of these results more precisely, further strengthening our conclusions.

## 5 CONCLUSIONS

Our meta‐analysis confirms that increasing outdoor time is an effective strategy for delaying the onset of myopia in children and adolescents. The findings support a significant reduction in axial elongation and a more favourable spherical equivalent in groups exposed to outdoor activities. However, variability in study methodologies suggests that factors such as light intensity and exposure duration influence outcomes. Based on existing evidence, we recommend at least 2 h of daily outdoor exposure, preferably with light intensities above 10,000 lux, to maximize protective effects against myopia. Integrating this into school and extracurricular routines could play a crucial role in reducing myopia incidence.

## ETHICAL APPROVAL

This article does not contain any studies with human participants or animals performed by any of the authors.

## Supporting information


Appendix S1.

Appendix S2.

Appendix S3.
