# Supplementary material for: Influence of outdoor time on the spherical equivalent and axial length in childhood myopia: A meta‐analysis
Source: Acta Ophthalmol. 2025 Mar 11;103(8):864–78. doi: 10.1111/aos.17478 (PMC12604451; doi:10.1111/aos.17478)
Supplement: Supplementary file 1 — Appendix S1. [file AOS-103-864-s001.docx]

PubMed search strategy.

Search: **("outdoor" OR "outside" OR "outdoor activity" OR "sunlight") AND ("myopia" OR "nearsightedness" OR "short sight" OR "refractive error") AND ("child" OR "children" OR "pediatric" OR "youth" OR "adolescent" OR "teenage") AND ("time" OR "hour*" OR "minute*")**

("outdoor"[All Fields] OR "outside"[All Fields] OR "outdoor activity"[All Fields] OR "sunlight"[All Fields]) AND ("myopia"[All Fields] OR "nearsightedness"[All Fields] OR "short sight"[All Fields] OR "refractive error"[All Fields]) AND ("child"[All Fields] OR "children"[All Fields] OR "pediatric"[All Fields] OR "youth"[All Fields] OR "adolescent"[All Fields] OR "teenage"[All Fields]) AND ("time"[All Fields] OR "hour*"[All Fields] OR "minute*"[All Fields])
