# Supplementary material for: Influence of outdoor time on the spherical equivalent and axial length in childhood myopia: A meta‐analysis
Source: Acta Ophthalmol. 2025 Mar 11;103(8):864–78. doi: 10.1111/aos.17478 (PMC12604451; doi:10.1111/aos.17478)
Supplement: Supplementary file 2 — Appendix S2. [file AOS-103-864-s002.docx]

Table 2. Conflict of Interest and Funding Information for Studies on Outdoor Interventions for Myopia Prevention

| **Study** | **COI**  **(Yes/No)** | **Funding**  **(Yes/No)** |
| --- | --- | --- |
| Guo et al. (2019) | No | Yes |
| He et al. (2015) | No | Yes |
| He et al. (2022) | No | Yes |
| Jin et al. (2015) | No | Yes |
| Liao et al. (2023) | No | Yes |
| Lin J et al. (2018) | No | Yes |
| Wu et al. (2013) | No | Yes |
| Wu et al. (2018) | No | Yes |
| Yi & Li (2011) | No | No |

COI: Conflict of interest

Table 3. Conflict of Interest and Funding Information for Observational Studies on the Association Between Outdoor Activities and Myopia Progression

| **Study** | **COI**  **(Yes/No)** | **Funding**  **(Yes/No)** |
| --- | --- | --- |
| Atowa, Wajuihian & Munsamy (2020) | No | Yes |
| Dhakal et al. (2024) | No | Yes |
| French et al. (2013) | No | Yes |
| Gopalakrishnan et al. (2023) | No | Yes |
| Jones et al. (2007) | No | Yes |
| Wen et al. (2020) | No | Yes |

COI: Conflict of interest
