# Supplementary material for: Influence of outdoor time on the spherical equivalent and axial length in childhood myopia: A meta‐analysis
Source: Acta Ophthalmol. 2025 Mar 11;103(8):864–78. doi: 10.1111/aos.17478 (PMC12604451; doi:10.1111/aos.17478)
Supplement: Supplementary file 3 — Appendix S3. [file AOS-103-864-s003.docx]

Risk of bias judgement.

Guo et al.2019

1. Potential limitations of our study should be mentioned. First, with only one school selected for each arm of the investigation, the recruitment of the study participants did not occur in a randomized manner.
2. Not reported
3. Not reported
4. Not reported
5. Not reported
6. All kind of outcomes.

He et al. 2015

1. Two schools were randomly selected from each of the strata,with 1 school allocated tothe interventionand 1 school allocated to the control. Schools in the intervention and control groups were matched closely in terms of longitudinal loss of visual acuity, which correlates closely with the development ofmyopia inchildren.This randomization process was performed with a simple random sampling using SAS version 9.2 (SAS Institute Inc), which generated 12 schools in 6 strata as the participating schools for the study. Cluster randomization by school was chosen as the study design because school-based interventions can change students’ behavior through mandatory changes in curriculum.
2. Figure. Flow of Participants in the Guangzhou Outdoor Activity Longitudinal Trial
3. Not reported
4. Not reported
5. Figure. Flow of Participants in the Guangzhou Outdoor Activity Longitudinal Trial
6. All kind of outcomes

He et al. 2022

1. The sample size was calculated based on the cluster-randomized design that accounted  for the intracluster correlation coefficient, the expected effect size, the power of the study, and the cluster size.
2. Figure 1.
3. Not reported.
4. Not reported.
5. Figure 1.
6. All kind of outcomes.

Jin et al. 2015

1. Figure 1.
2. Figure 1.
3. Not reported.
4. Not reported.
5. Figure 1.
6. All kind of outcomes.

Liao et al. 2023

1. We conducted a randomized controlled trial on the effects of outdoor light and exercise intervention on children’s vision in a primary school in Chengdu from March 2022 to March 2023.
2. Figure 1.
3. Not reported.
4. Not reported.
5. Figure 1.
6. All kind of outcomes.

Lin et al. 2018

1. Three primary schools in Wenzhou area... Three schools were randomly assigned to perform different outdoor interventions
2. Not reported
3. The teachers supervised the students' outdoor activities during recess.
4. Ophthalmic A/B ultrasound was used to measure axial length.
5. Not reported.
6. All kind of outcomes.

Wu et al. 2013

1. Not reported.
2. Not reported.
3. Not reported.
4. Not reported.
5. Not reported.
6. All kind of outcomes.

Wu et al. 2018

1. We conducted a multi-area cluster-randomized controlled trial for myopia prevention from September 2013 through February 2015.
2. Figure 1.
3. Measurements were performed by ophthalmologists and trained research assistants who were blinded to intervention conditions.
4. Not reported.
5. Figure 1.
6. All kind of outcomes.

Yi Jun-Hui et al. 2011

1. Eighty schoolchildren aged 7-11 with myopia were randomly assigned to an intervention group (41 children) and a control group (39 children).
2. Not reported.
3. Not reported.
4. The degree of myopia progression was measured via regular refraction exams over two years.
5. A total of 10 children in the control group were lost to follow-up, leaving 29 in the study.
6. All kind of outcomes.
